# Saccades Influence the Visibility of Targets in Rapid Stimulus Sequences: The Roles of Mislocalization, Retinal Distance and Remapping

**DOI:** 10.3389/fnsys.2016.00058

**Published:** 2016-06-28

**Authors:** Alessio Fracasso, David Melcher

**Affiliations:** ^1^Experimental Psychology, Helmholtz Institute, Utrecht UniversityUtrecht, Netherlands; ^2^Center for Mind/Brain Sciences, Department of Cognitive Sciences, University of TrentoRovereto, Italy

**Keywords:** eye movements, perisaccadic perception, forward masking, mislocalization, rapid serial visual presentation

## Abstract

Briefly presented targets around the time of a saccade are mislocalized towards the saccadic landing point. This has been taken as evidence for a remapping mechanism that accompanies each eye movement, helping maintain visual stability across large retinal shifts. Previous studies have shown that spatial mislocalization is greatly diminished when trains of brief stimuli are presented at a high frequency rate, which might help to explain why mislocalization is rarely perceived in everyday viewing. Studies in the laboratory have shown that mislocalization can reduce metacontrast masking by causing target stimuli in a masking sequence to be perceived as shifted in space towards the saccadic target and thus more easily discriminated. We investigated the influence of saccades on target discrimination when target and masks were presented in a rapid serial visual presentation (RSVP), as well as with forward masking and with backward masking. In a series of experiments, we found that performance was influenced by the retinal displacement caused by the saccade itself but that an additional component of un-masking occurred even when the retinal location of target and mask was matched. These results speak in favor of a remapping mechanism that begins before the eyes start moving and continues well beyond saccadic termination.

## Introduction

Saccadic eye movements are ballistic eye movements aimed to reposition the most sensitive area of the retina, the fovea, to receive information about targets of interest. These movements can be voluntary but tend to go unnoticed during daily life. Saccades are accompanied by a large variety of perceptual effects, including suppression of the visual input (Dodge, 1900; Latour, 1962; Macknik et al., 1991), suppression of saccadic target displacement (Deubel et al., 1996), mislocalization of briefly presented targets around the time of the saccades (Ross et al., 1997) and even time compression/inversion (Morrone et al., 2005; Binda et al., 2009).

Neurophysiological studies have reported the existence of neurons that show receptive field (RF) shifts around the time of saccades (Duhamel et al., 1992; Zirnsak et al., 2014), with the response of retinotopic neurons gradually shifting from the current RF to the future RF (the position in space occupied by the RF after the completion of the eye movement, Kusunoki and Goldberg, 2003). In some brain regions, such as the Frontal Eye Fields (FEF) and area V4, RFs seem to shift or compress towards the saccadic target location (Tolias et al., 2001; Zirnsak et al., 2014). This shift, referred to as “remapping”, anticipates the start of the actual eye movement and evolves gradually, starting well before eye movement onset, and continues also when the eyes are actually moving. This shift is interpreted as a predictive signal that anticipates the outcome of the incoming eye movement (Melcher and Fracasso, 2012).

Studies investigating the effect of eye-movements on visual masking, using a pre-saccadic mask and a post-saccadic target, have reported that it is largely a retinotopic phenomenon (Irwin et al., 1988). Recently, the interaction between eye movement and metacontrast masking sequences has been studied with a crucial difference with respect to previous investigations: performance was measured also during the pre-saccadic interval, when target and mask fell in contiguous retinal coordinates (De Pisapia et al., 2010). De Pisapia et al. (2010) showed that briefly presented targets in a metacontrast masking paradigm can be “unmasked” if the targets and mask are presented during the perisaccadic interval. Participants reported the *target* as mislocalized towards the future saccadic landing position, rather than at the same position as the subsequent metacontrast mask. Thus, the target identity could be more easily reported, leading to increased performance. These results have been interpreted as reflecting a remapping process accompanying the execution of the eye movement, since discrimination performance increased for targets that were reported as mislocalized compared to non-mislocalized trials.

In everyday life, however, mislocalization around the time of saccades seems to be rare or non-existent. One likely explanation is that stimuli are not usually flashed briefly in the peri-saccadic time period. Objects tend to be stable over time, rather than suddenly appearing and disappearing. Previous studies have shown that spatial mislocalization is reduced when targets are shown for longer time periods or by presenting trains of flashes of stimuli, rather than a single briefly flashed stimulus, which might help to integrate the stream of stimuli into a unique event that span the duration of a saccade (Honda, 2006).

Based on these findings, we presented targets and masks in a rapid serial visual presentation (RSVP) stream in order to test whether unmasking occurs also without spatial mislocalization or, instead, whether perceptual mislocalization is necessary to obtain the improvement in discrimination performance observed in the saccade unmasking paradigm. In the first step (Experiment 1) we determine that discrimination performance on the RSVP task improved for a rapid series of alternating targets and masks increased in the peri-saccadic time period with respect of stable fixation. Furthermore, we obtained estimates of the facilitation in the RSVP when the participants were required to perform an eye-movement compared to fixation. In Experiment 2 we analyze the temporal specificity of discrimination performance around the perisaccadic interval, in order to control whether the facilitation observed in Experiment 1 could be ascribed just to the retinal separation between targets and masks while the eyes were moving towards the fixation landing point.

The results of Experiment 2 indicated that most of the masking power could be ascribed to the forward mask and that retinal separation between the target and the forward mask played a crucial role in the improvement in performance over the RSVP task in Experiment 1. In Experiment 3 we focused on the main driver of the RSVP masking sequence, forward masking, and asked whether mislocalization could improve discrimination performance, exceeding the advantage expected solely by the retinal separation.

Findings on the third experiment indicated that perisaccadic mislocalization was related to improved performance, exceeding the benefit given by retinal distance, mainly for brief interstimulus interval (ISI) durations. Overall, measuring discrimination performance, the retinal distance between target and mask and the subjective reported mislocalization along the perisaccadic interval in Experiment 3, allowed us to test whether predictive remapping evolves gradually, before eye movement onset, and continuously, also while the eyes are moving towards saccade landing position.

## General Methods

### Participants

A total of 11 participants took part in the three experiments (5 women, mean age, 28.6 years; range, 22–32 years), four participants in Experiment 1, five participants on Experiment 2 and four participants on Experiment 3. All subjects had normal or corrected-to-normal vision. Participants were paid for their participation. The experiments were conducted in accordance with the ethical guidelines for psychophysical studies laid down by the University of Trento and the ethical standards laid down in the 1964 Declaration of Helsinki (most recently amended in 2008, Seoul). All participants were naïve with regard to the main hypothesis of the experiment except for one author who participated in the study as a subject. Written informed consent was obtained from all subjects.

### Experimental Setup

Observers sat in a dimly lit room and viewed the computer screen at a distance of 57 cm with their heads supported on a chin rest. Eye movements were measured using an EyeLink 1000 Desktop Mount (SR Research, Toronto, ON, Canada) sampling at 1 kHz. Software implemented in MATLAB (MathWorks, Natick, MA, USA) controlled stimulus display and response collection using the Psychophysics toolbox (Brainard, 1997) and EyeLink toolbox (Cornelissen et al., 2002). Stimulus sequences were presented on an Iiyama CRT 1900 monitor (1280 columns × 1024 lines, refresh rate: 85 Hz for Experiment 1 and 100 Hz for Experiments 2 and 3) on a uniformly gray background with an average luminance of 8.8 cd/m^2^ (CIE coordinates: *x* = 0.28; *y* = 0.31).

**Figure 1:**
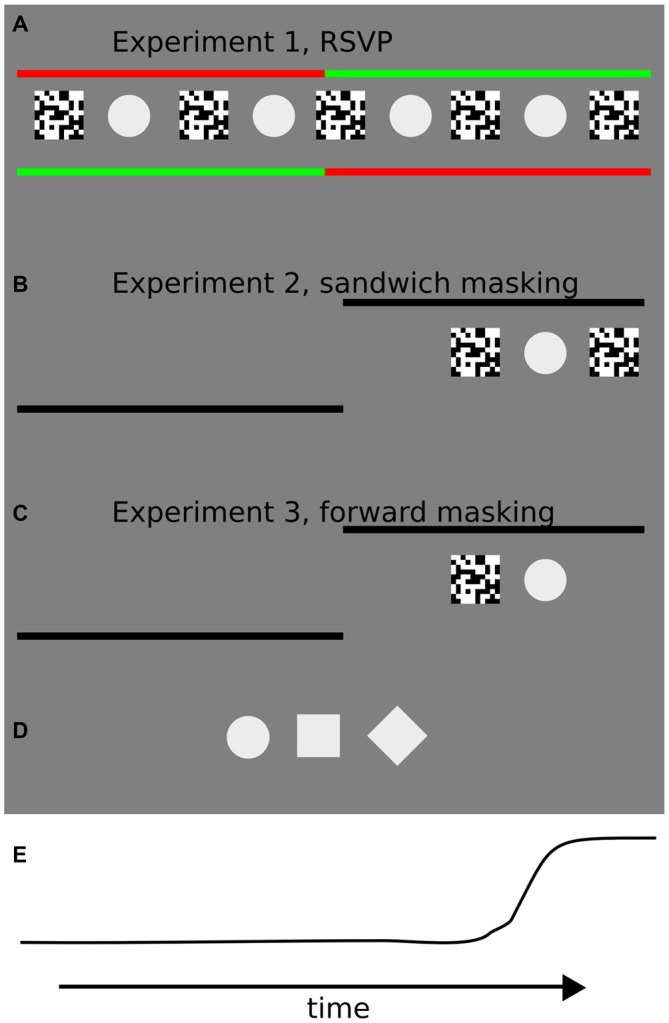
**Illustrations of the trial sequences for each of the three experiments in the study. (A)** In Experiment 1, participants were instructed to keep fixation on the starting (green) fixation point until it changed color, and then to perform a 10° of visual angle saccade towards the peripheral fixation point. Starting and landing fixation points were shifted each for 5° of visual angle with respect to the screen center. In the center of the screen the rapid serial visual presentation (RSVP) sequence was presented at different interstimulus intervals (ISIs). Starting and landing fixation points (left or right) were randomly assigned on each trial. **(B)** In Experiment 2, participants were instructed to keep fixation on the starting fixation point until it disappeared, and then to perform a 10° of visual angle saccade towards the peripheral fixation point. The starting fixation point was located at the center of the screen and the side of the landing fixation point (hence the direction of the saccade, left or right) was randomly assigned prior to each trial. The “sandwich masking” sequence was presented after a random interval after the appearance of the landing fixation point at 5° of visual angle from the center of the screen, on the side marked by the landing fixation point. **(C)** In the third experiment, the procedure was identical to Experiment 2, except that a forward masking sequence was adopted. The forward masking sequence was presented after a random interval after the appearance of the landing fixation point. **(D)** Target stimuli used in the three experiments: circle, square and diamond. Contrast is enhanced for visualization purposes. During the experiments, the stimulus contrast was set to 7%. **(E)** An example of an eye movement trajectory (horizontal position).

The target stimulus on each trial was one of three different shapes (Figure 1D), a circle, (diameter ~2.82°/visual angle, area ~6.26 (°/visual angle)^2^), a square (side ~2.5°/visual angle, area ~6.25 (°/visual angle)^2^) or a diamond based on the previous square rotated by 45°. Masks consisted of noise patterns (side ~4.4°/visual angle) composed by black (CIE coordinates: *x* = 0.35, *y* = 0.37; luminance = 0.25 cd/m^2^) and white (CIE coordinates: *x* = 0.28; *y* = 0.30, luminance: 80 cd/m^2^) squares (0.058°/visual angle each), on each trial one or multiple random noise masks were generated, depending on the ongoing experiment (Experiment 1: 34 masks were generated; Experiment 2: two different masks were generated; Experiment 3: one mask was generated).

### Eye Movements

Before each session, a five-point calibration routine was run and drift correction was applied. Throughout the session (each block in the experiments comprised 50 trials), drift correction was run five times. Prior to the analysis, saccade size and latency were analyzed for each trial. Trials were excluded when the saccade performed was too short (<7° of visual angle), or latencies were >500 ms or <100 ms. With these criteria ~10% of the trials were discarded in each of the following experiments.

## Experiment 1: Unmasking the Target in an RSVP Masking Paradigm

Each trial began with two fixation points (0.29°/visual angle diameter) placed on opposite sides of the screen, each 5° of visual angle to the right or left (10° apart from each other). One fixation point was red (CIE coordinates: *x* = 0.62; *y* = 0.30) and one green (CIE coordinates: *x* = 0.28; *y* = 0.54), with the color order randomized on each trial (Figure 1A).

Participants were instructed to fixate the green fixation point and press a button when they felt ready to start the trial. After the button press a RSVP of target and random masks were presented at a variable alternation rate.

On each trial the RSVP consisted of 34 mask + target repetitions. During the first six repetitions, target contrast was ramped up linearly from 1% to 6% contrast until reaching the final test level of 7% contrast, followed by 22 repetitions at test contrast level and then ending with six mask arrays dropping linearly in contrast (from 6% till 1% contrast). The linear increase and decrease of target contrast avoided a sudden onset or termination that could allow successful identification even at very high alternation rates (Beaudot, 2002).

Each RSVP sequence started and ended with the presentation of a noise mask. Target and masks remained on the screen for ~22 ms (two frame refreshes at 85 hz). The alternation rate was changed by varying the ISI between target and mask (Cavanagh et al., 2008).

After 17 mask + target repetitions the colors of the fixation points changed, signaling participants to perform a saccade towards the new green fixation point (10° of visual angle to the left or right with respect to the starting fixation point, Figure 1E) on those blocks in which they were instructed to saccade. On no saccade blocks, participants were required to maintain fixation on the initial fixation point even when the color abruptly changed. The viewing condition (saccade or fixation) was varied across blocks and the overall order of blocks was counterbalanced across subjects to avoid order effects.

Six levels of ISI were randomly presented across trials (11, 33, 55, 77, 99, 198 ms). After each trial the screen was blanked for 500 ms and then subjects were requested to report the identity of the presented target or to guess otherwise.

Responses were given using keys 1, 2 and 3 on a keypad (“circle”, “diamond” and “square”, respectively, in a 3-alternative forced-choice, AFC task), with a reminder of the key-to-shape mapping presented after each trial. Participants had unlimited time to provide a response after the trial. Four participants were presented with 24 blocks of 12 trials each, for a total of 288 trials. Participants were tested in two non-consecutive days.

## Experiment 1: Pilot Experiment

A short pilot experiment was run prior to Experiment 1 where we tested the same participants on the same apparatus in the saccade condition with three different ISIs (11, 33, 55 ms). Participants were asked to report whether they perceived perceptual mislocalization of targets or masks towards saccade landing fixation point.

Responses were given using keys 1, 2 on a keypad (“yes”, “no”). Participants had unlimited time to provide a response after the trial. Four participants were presented on 150 trials, 50 trials for each ISI tested.

## Experiment 1: Data Analysis

Data from the four participants was pooled together and analyzed fitting a Weibull function following the parameterization reported in Kuss et al. (2005), see Equation 1. The model was fit on each iteration via the R software implementation of non-linear least squares on 2000 bootstrap dataset repetitions (with replacement) and the 95% confidence interval (CI) of each of the slope and threshold (thr) parameters was estimated.

(1) $$\begin{array}{l} p\left(slope,thr,ISI\right)\;\\ \;=1-\exp(-\exp\left(\frac{2*slope*thr}{\ln\left(2\right)}\right)\\ \;*\left(\ln\left(ISI\right)-\ln\left(thr\right)\right)+\ln\left(\ln2\right)\text{ ​}) \end{array}$$

## Experiment 1: Results and Discussion

**Figure 2:**
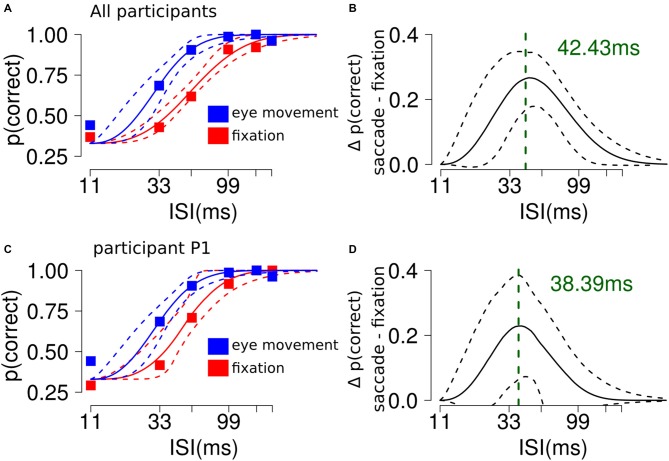
**The influence of saccades on RSVP masking in Experiment 1. (A)** Participants showed better discrimination performance in the saccade condition compared to fixation. The plot shows the average performance for each ISI condition and the 95% confidence interval (CI) of the Weibull fit. **(B)** The analysis shows a significant difference along ISI levels between the saccade and fixation conditions. The plot shows a curved trend and indicates that the largest difference between the experimental conditions could be found at ~40 ms. **(C,D)** Results from a single representative participant.

Participants showed better discrimination performance in the saccade condition (95% CI thr = [24, 37] ms) compared to fixation (95% CI thr = [48, 63] ms, Figures 2A,C). The slope parameter did not differ between the conditions. To characterize the difference between the two curves we subtracted the discrimination values estimated from the model in the saccade and fixation conditions, across ISI. This resulted in a curve with a peak located at the theoretical ISI with the largest difference in performance between the experimental conditions (peak location = ~42 ms, see Figures 2B,D).

Results of the pilot experiment showed that participants did not report any perceptual mislocalization during saccade trials. The average proportion of trials with reported mislocalization across participants was 1%, 5% and 3% for the 11, 33 and 55 ms condition, respectively.

These results indicate that performing an eye movement during an RSVP sequence of target-mask stimuli “unmasks” the target. This raises the question of whether the effect was driven by peri-saccadic effects such as have been shown previously in mislocalization and masking (De Pisapia et al., 2010; Fracasso et al., 2015) paradigms, or was based exclusively on the saccade itself changing the retinal position of target with respect to mask. To clarify these issues we ran Experiment 2.

## Experiment 2: Sandwich Masking, Methods

### Experimental Condition: Eye Movement Trials

Each trial began with a single black (CIE coordinates: *x* = 0.35, *y* = 0.37; luminance = 0.25 cd/m^2^) fixation point (0.29°/visual angle diameter) placed in the center of the screen. Participants were instructed to fixate and press a button when they felt ready to start the trial. After a variable delay between 750 and 1250 ms the starting fixation point disappeared and a second black landing fixation point appeared either to the left or the right of the screen (10° of visual angle to the left or right with respect to the central starting fixation point, randomized across trials). Participants were instructed to perform a saccade as fast as possible towards the landing fixation point. At different timings with respect to saccade onset, a sandwich masking sequence was presented, consisting of a forward noise mask, a target and a subsequent backward noise mask (Kaunitz et al., 2014). Please note that the forward noise mask and the backward noise mask shared pixels on the screen with the target (Figure 1B), contrary to the “standing wave illusion” which uses non-overlapping stimuli, introduced by Macknik and Livingstone (1998), and subsequently studied by Macknik and Haglund (1999), Macknik et al. (2000), Macknik and Martinez-Conde (2004) and Tse et al. (2005). Targets could be either a circle, a square or a diamond at 5% contrast (3AFC task). The sandwich masking sequence was presented between the starting and the landing fixation points, at 5° of visual angle eccentricity. The ISI between masks and stimuli was either 30 or 40 ms, constant for each masking sequence. Target and masks remained on the screen for 20 ms (2 frame refreshes).

### Control Conditions

Two separate control conditions were run at fixation on the same participants on separate, non-consecutive days, The only procedural difference between experimental and control conditions was that the starting fixation point always remained visible and a landing fixation point never appeared in the periphery, hence the masking sequence could randomly appear on the left or right hemifield while participants fixated at the screen center.

In the first control condition, two blocks of 50 trials were run for each participant. The masking sequence consisted on the entire sandwich masking sequence (forward mask—target—backward mask) randomly presented in the left or right hemifield with an ISI between masks and target of 30 or 40 ms. The ISI on each trial was randomized.

In the second control condition, four 50 trials blocks were run for each participant. Two different conditions were tested in a 2 × 2 design: the masking sequence could consist of either forward only or backward only mask, and ISI could be either 30 or 40 ms. On each trial the experimental condition was randomly selected and the masking sequence with ISI interval were randomly presented in the left or right hemifield.

## Experiment 2: Data Analysis

### Behavioral Performance: Eye Movement Trials

Data from the five participants were pooled together and analyzed with a logistic regression model with the formulation proposed by McCullagh and Nelder (1989). The data was binned into 10 different perisaccadic intervals, according to 10% percentile of target onset time with respect to saccade onset, in order that each bin contained the same number of trials. The following equation was fit to the data:

(2) $$y=\beta_{0}+perisaccadicinterval_{1..9}*\beta_{1..9}$$

This is a convenient formulation since the estimated model parameters represented the difference in discrimination accuracy with respect to the baseline perisaccadic interval. The first perisaccadic interval was taken as baseline in our model. The model was fit on each iteration of 2000 bootstrap dataset repetitions (with replacement) and the 95% CI of each of the 10 parameters of Equation 2: β_0_ to β_9_. Significance was assessed by comparing the CI of the first perisaccadic interval with the CI derived from the remaining perisaccadic intervals. We also fit the data using a multi-level linear model, modeling separately the contribution of each single participant with a separate intercept (Gelman and Hill, 2006).

### Retinal Distance Computation

The retinal distance between the target and the forward or backward masks of the sandwich masking sequence was computed on each trial to evaluate its influence on discrimination performance. We extracted single-trial eye position traces. From these traces, we (i) computed the median eye position during the time interval from target onset until target termination; and (ii) computed the median eye position during the time interval from forward (backward) mask onset time until forward (backward) mask termination. Then we subtracted these two values to obtain target—forward (backward) mask retinal distance and we took the absolute value to obtain the distance between target and forward (backward) mask on the retina (Fracasso et al., 2013, 2015). The forward (backward) mask retinal distance was divided into 10 bins, according to the 10% quantiles of the forward (backward) mask distribution. Discrimination performance was computed for each of the forward (backward) mask retinal distances. The relation between discrimination accuracy and forward (backward) mask retinal distance bin was assessed fitting a general linear model and using a multi-level linear model, modeling separately the contribution of each single participant with a separate intercept, accounting for the perisaccadic interval covariate (Gelman and Hill, 2006). The observed and predicted trials were binned into 20 separate bins and *p*(correct) was computed for each bin. The agreement between the model and the observed data was tested by linear regression.

### Behavioral Performance, Fixation

For the first control condition, the data from the five participants was pooled together and discrimination performance was tested against the null hypothesis of being at chance level (*p*(discrimination)_HO_ = 0.33) via a test on proportions.

For the second control condition, the data from the five participants was pooled together and analyzed with a logistic regression model with the formulation proposed by McCullagh and Nelder (1989), fitting the following equation:

(3) $$\begin{array}{l} y=\beta_{0}+ISI*\beta_{1}+maskingcondition*\beta_{2}\\ \;+ISI*maskingcondition*\beta_{3} \end{array}$$

As described above, the linear combination of the estimated model parameters represented the discrimination average for each of the four experimental conditions tested. The model was fit on each iteration of 2000 bootstrap dataset repetitions (with replacement) and the 95% CI derived. Significance was assessed by comparing the derived CI of the experimental conditions.

## Experiment 2: Results and Discussion

**Figure 3:**
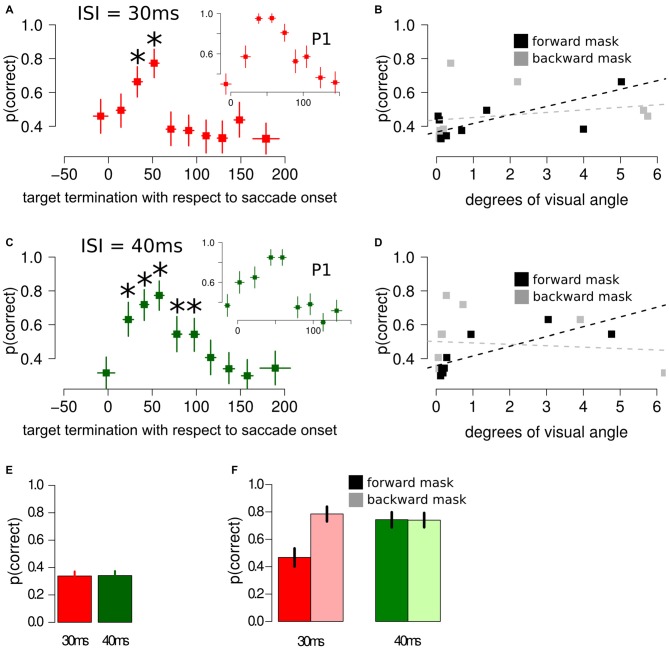
**Results of Experiment 2, using different masking sequences. (A)** Discrimination performance around the perisaccadic interval for sandwich masking with an ISI of 30 ms. The inset represent the perisaccadic performance from a single participant, P1. Horizontal error bars represent one sd of target termination with respect to saccade onset for the corresponding bin. Vertical error bars represent Bonferroni corrected 95% CI of the logistic repression estimate of the corresponding bin. The asterisks indicate a significant difference with respect to the performance in the pre-saccade onset bin. **(B)** Forward mask—target stimuli retinal distance and backward mask—target stimuli retinal distance were computed on each bin. Forward mask—target stimuli retinal distance reliably predicted discrimination performance (black lines and dotted line), whereas no significant trend was found for backward mask—target stimuli predictor (gray lines and dotted line). **(C)** Discrimination performance and CIs around the perisaccadic interval for the sandwich masking with an ISI of 40 ms. The inset represent the perisaccadic performance from a single participant, P1. **(D)** Also with an ISI of 40 ms, forward mask—target stimuli retinal distance was able to reliably predict discrimination performance. **(E)** Sandwich-masking performance at fixation for 30 ms and 40 ms ISI. Error bars represent two SEMS. **(F)** Discrimination performance at fixation for forward mask only (full color) and backward mask only (light color) with an ISI of 30 ms (red bars) and 40 ms (green bars). Error bars represent 95% CIs performance estimates in the corresponding condition, derived from a logistic regression model. Please note that the representation in **(B,D)** is relatively simple and aimed at visually appreciating the association between forward retinal distance and *p*(correct). This representation does not take into account target termination with respect to saccade onset, a crucial covariate, which is taken into account by the multi-level model, (see “Experiment 2: Results and Discussion” Section and Figure 4).

When asked to perform an eye movement while being presented with a sandwich masking sequence with an ISI of 30 ms, participants showed an increased discrimination performance during the perisaccadic intervals (23, 42] ms (*p*(discrimination) = 0.66) and (42, 61] ms (*p*(discrimination) = 0.77), compared to the first perisaccadic interval (−47, 3] (*p*(discrimination) = 0.46), see Figure 3A. The same results were obtained fitting a multi-level linear model on the data: (23, 42] ms, *z* = 2.859, *p* < 0.005 and (42, 61] ms, *z* = 4.407, *p* < 0.001. Retinal distance analysis revealed that the forward mask—target retinal distance was a very accurate predictor of discrimination performance (*t* = 4.192, *p* = 0.003), whereas backward mask—target retinal distance did not predict discrimination performance in the perisaccadic interval (*t* = 0.675, *p* = 0.519), see Figure 3B.

Comparable results were obtained fitting a multi-level linear model, with different participants modeled with different intercepts, including the perisaccadic intervals as covariates: the forward mask—target retinal distance was an accurate predictor of discrimination performance (*z* = 1.772, *p* = 0.065), whereas backward mask—target retinal distance did not predict discrimination performance in the perisaccadic interval (*z* = −0.740, *p* = 0.459).

When asked to perform an eye movement while being presented with a sandwich masking sequence with an ISI of 40 ms, participants showed an increased discrimination performance during the perisaccadic intervals (12, 32] ms (*p*(discrimination) = 0.63), (32, 49] ms (*p*(discrimination) = 0.72), (49, 67] ms (*p*(discrimination) = 0.77), (67, 86] ms (*p*(discrimination) = 0.54) and (86, 106] ms (*p*(discrimination) = 0.54), compared to the first perisaccadic interval (−36, 12] (*p*(discrimination) = 0.31), see Figure 3C. The same results were obtained fitting a multi-level linear model on the data: (12, 32] ms, *z* = 4.144, *p* < 0.001; (32, 49] ms, *z* = 5.190, *p* < 0.001; (49, 67] ms, *z* = 6.083, *p* < 0.001; (67, 86] ms, *z* = 2.985, *p* < 0.005 and (86, 106] ms *z* = 2.993, *p* < 0.005. Retinal distance analysis reveals that the forward mask—target retinal distance was a very accurate predictor of discrimination performance (*t* = 6.462, *p* < 0.001), whereas backward mask—target retinal distance did not predict discrimination performance in the perisaccadic interval (*t* = −0.287, *p* = 0.782), see Figure 3D.

Comparable results were obtained fitting a multi-level linear model, with different participants modeled with different intercepts, including the perisaccadic intervals as covariates: the forward mask—target retinal distance was a very accurate predictor of discrimination performance (*z* = 2.337, *p* = 0.019), whereas backward mask—target retinal distance did not predict discrimination performance in the perisaccadic interval (*z* = −1.113, *p* = 0.265).

**Figure 4:**
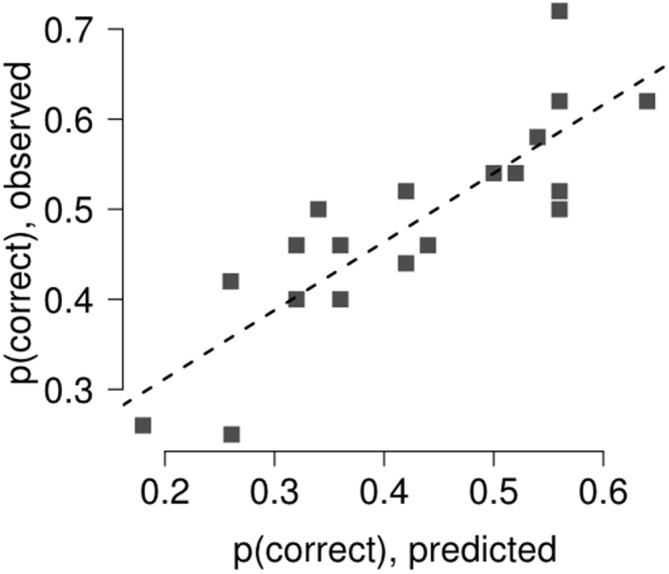
**The relation between discrimination accuracy and forward (backward) mask retinal distance bin was assessed fitting a general linear model and using a multi-level linear model, modeling separately the contribution of each single participant with a separate intercept, accounting for target termination with respect to saccade onset (Gelman and Hill, 2006).** The agreement between the model and the observed data was tested by linear regression. The model captured most of the variance in Experiment 2 (*R*^2^ = 72%).

Results of the multi-level linear model with the comparison between predicted and observed discrimination accuracy are reported in Figure 4. The relationship between predicted and observed discrimination accuracy was tested using a general linear model, showing good agreement between the variables, with a variance explained above 70% (*R*^2^ = 72%, *t* = 6.73, *p* < 0.0001).

When asked to keep fixation while being presented with a sandwich masking, participants’ performance was not different than chance (*p*(discrimination) = 0.33) when the ISI was set to 30 ms (*χ*^2^ = 0.030, *p* = 0.860) and was not different than chance when the ISI was set to 40 ms (*χ*^2^ = 0.075, *p* = 0.783), see Figure 3E.

When asked to keep fixation while being presented with either a forward or backward masking sequence, performance was selectively impaired only when participants were presented with a forward masking sequence with an ISI of 30 ms, whereas the remaining three experimental conditions did not differ between each other, see Figure 3F.

These results indicate that discrimination performance was increased by performing an eye movement over a sandwich masking sequence mainly when the target was presented right after the saccade onset, with a greater performance increase when the ISI was set to 40 ms compared to 30 ms. This discrimination performance increase could be well explained by the forward mask—target retinal distance around the perisaccadic interval, and the results on the second control experiment, at fixation, support this result since a significant performance decrease using noise masks could be found only in the forward masking condition.

However, one aspect of this perisaccadic discrimination increase still needs to be clarified. A crucial determinant in perisaccadic unmasking using backward masking sequences is perisaccadic mislocalization of the target. Previous research has shown that discrimination performance dramatically increases on those trials where the target is perceived as mislocalized with respect to the mask (De Pisapia et al., 2010; Fracasso et al., 2015). In the present experiment we used a sandwich masking sequence that encompassed the duration of the eye movement and no masks or target mislocalization was reported by the participants, as could be expected based on previous research using transient stimuli encompassing the duration of the eye movement (Honda, 2006).

In Experiment 3 we investigated the role of perisaccadic unmasking on forward mask sequences. With this kind of masking sequence the mask in presented before the target, we expect the mask itself to be reported as mislocalized, not the target as found in previous studies using backward or metaconstrast masking (De Pisapia et al., 2010; Fracasso et al., 2015). Moreover, this new experiment tested whether retinal distance would also play a major role also in the case of an unmasking performance increase after saccade onset, or if it were possible to find a dissociation between the role of retinal distance and reported mislocalization.

## Experiment 3: Mask Mislocalization, Methods

The trial procedure was identical to Experiment 2 with the following differences: (i) only one masking sequence was adopted, namely forward masking (forward mask—target, Figure 1C); (ii) after each trial, two different questions were asked to the participant, the first was to report the identity of the stimuli presented if perceived or to guess otherwise (3AFC), as in the previous experiments. In the second question participants were asked to report the perceived location of the mask with respect to the target stimuli using keys 1, 2 and 3 on a keypad (“left to the target”, “same location as the target”, “right to the target”). A reminder of the question was presented after each trial. Participants were instructed to report “same location as the target” in case they did not perceive the target.

Three different versions of the same experiment were run, changing masking parameters. In the first version, the ISI was set to 30 ms and both noise mask and the stimuli remained on the screen for 20 ms (2 frame refreshes). For the second version, the ISI was set to 10 ms and both noise mask and the target remained on the screen for 10 ms (1 frame refreshes). In the third and last version ISI was set to 0 ms (no ISI was employed) and both noise mask and the target remained on the screen for 10 ms (1 frame refreshes), presented subsequently one after the other. As with Experiment 2, the target was presented between the starting and the landing fixation points, at 5° of visual angle eccentricity. Each participant took part on the three versions of the experiment. On each version, a variable number of 50 trials blocks was performed by each participant, for a total number of trials that ranged from 400 to 600.

Masks were coded as mislocalized on those trials in which the reported location of the mask with respect to the target stimuli was congruent with saccade direction (e.g., saccade requested to the left, mask reported to be perceived to the left of the target stimuli), consistently with mislocalization reports of briefly flashed targets presented in the middle of a saccade trajectory (Matin, 1974; Honda, 1985; Ross et al., 1997).

## Experiment 3: Data Analysis

### Behavioral Performance, Eye Movement

For each of the three experiment versions, the data from the participants was pooled together and analyzed with a logistic regression model with the formulation proposed by McCullagh and Nelder (1989), data was binned on four different perisaccadic intervals form 50 ms before saccade onset (−50 ms) until 70 ms after saccade onset (70 ms). The following equation was fit to the data:

(4) $$\begin{array}{l} y=\beta_{0}+mislocalizationreport*\beta_{1}\\ \;+perisaccadicinterval*\beta_{2..4}\\ \;+mislocalizationreport*perisaccadicinterval*\beta_{5..7} \end{array}$$

As above, the model was fit on each iteration of 2000 bootstrap dataset repetitions (with replacement) and the 95% CI of each of the eight parameters of Equation 4: β_0_ to β_7_. Data was fit also using a multi-level linear model, modeling separately the contribution of each single participant with a separate intercept (Gelman and Hill, 2006). Significance was assessed by comparing the derived CI of the experimental conditions, a difference between two conditions is reported as significant only if it survived on the general linear model *and* the multi-level linear model (Bonferroni corrected).

### Retinal Distance Computation

The forward mask—target retinal distance was computed for each trial, as described above in Experiment 2. For each of the three experiment conditions the retinal distance data from the participants was pooled together and analyzed with a general linear model with the formulation proposed by McCullagh and Nelder (1989), using the same binning adopted for the discrimination accuracy analysis and the same equation (Equation 4) was fit to the retinal distance data. The only exception was that in the former case the link function was the logit (as in the logistic regression case), whereas in this case the identity function was used. The model was fit on each iteration of 2000 bootstrap dataset repetitions (with replacement) and the 95% CI of each of the eight parameters of Equation 4: β_0_ to β_7_.

In this way we could assess, for the same binning conditions, whether any influence of reported mislocalization on discrimination performance could be expected given the retinal distance between the forward mask and the target. As described above, the data was also fit using a multi-level linear model.

## Experiment 3: Results and Discussion

### ISI = 30 ms

**Figure 5:**
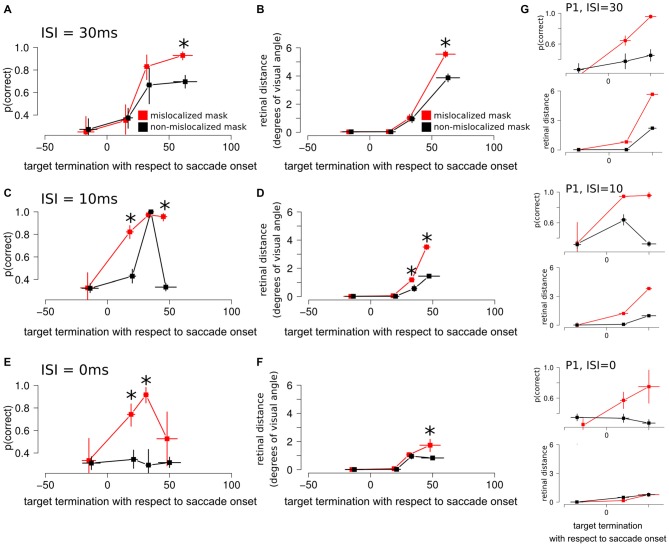
**Experiment 3 results. (A)** Discrimination performance around the perisaccadic interval for the forward masking with an ISI of 30 ms on mislocalized and non-mislocalized trials. The asterisks indicate a significant difference. **(B)** Retinal distance around the perisaccadic interval for the forward masking with an ISI of 30 ms on mislocalized and non-mislocalized trials. **(C)** Discrimination performance around the perisaccadic interval for the forward masking with an ISI of 10 ms on mislocalized and non-mislocalized trials. **(D)** Retinal distance around the perisaccadic interval for the forward masking with an ISI of 10 ms on mislocalized and non-mislocalized trials. **(E)** Discrimination performance around the perisaccadic interval for the forward masking with an ISI of 0 ms on mislocalized and non-mislocalized trials. **(F)** Retinal distance around the perisaccadic interval for the forward masking with an ISI of 0 ms on mislocalized and non-mislocalized trials. **(G)** Results reported on a single participant (P1) across all conditions.

When asked to perform an eye movement while being presented with a forward masking sequence with an ISI = 30 ms, participants showed a monotonic increase of discrimination performance along the perisaccadic intervals from (−50, 0] ms till (40, 70] ms on trials in which the forward mask was reported as non-mislocalized as well as on trials when the forward mask was reported as mislocalized (Table 1). A significant difference between discrimination performance on trials reported as mislocalized with respect of trials reported as non-mislocalized was found only for the perisaccadic interval (40, 70], see Figures 5A,B,G and Table 1.

**Table 1:** **95% confidence intervals derived from the bootstrapped analysis on *p*(correct) and retinal distance data from 30 ms ISI experiment**.

ISI = 30 ms	p(correct) (%)				retinal distance (degrees/vis. angle)			
Mislocalized	[0.12, 0.37]	[0.22, 0.49]	[0.72, 0.92]	**[0.88, 0.96]**	[0.02, 0.03]	[0.03, 0.05]	[0.73, 1.19]	**[4.99, 5.45]**
Non-mislocalized	[0.17, 0.36]	[0.28, 0.46]	[0.48, 0.80]	**[0.64, 0.75]**	[0.02, 0.06]	[0.03, 0.04]	[0.61, 1.19]	**[3.36, 3.90]**
Perisaccadic interval	(−50, 0]	(0, 20]	(20, 40]	(40, 70]	(−50, 0]	(0, 20]	(20, 40]	(40, 70]

*Data reported for each time bin along saccade onset time. Significant differences between mislocalized and non-mislocalized trials in the *p*(correct) and retinal distance variables are reported in bold*.

Retinal distance results closely mimicked those of discrimination performance. Participants showed a monotonic increase of discrimination performance along the perisaccadic intervals from (−50, 0] ms till (40, 70] ms on trials in which the forward mask was reported as non-mislocalized as well as on trials were the forward mask was reported as mislocalized (Table 1). A significant difference between discrimination performance on trials reported as mislocalized with respect of trials reported as non-mislocalized was found only for the perisaccadic interval (40, 70], see Figures 5A,B and Table 1. Average proportion of trials where the mask was reported as mislocalized with respect to the target was: 30%, 32%, 60% and 44% for the (−50, 0], (0, 20], (20, 40] and (40, 70] perisaccadic interval, respectively.

### ISI = 10 ms

When asked to perform an eye movement while being presented with a forward masking sequence with an ISI = 10 ms, participants showed a monotonic increase of discrimination performance along the perisaccadic intervals from (−50, 0] ms till (40, 70] ms on trials in which the forward mask was reported as mislocalized (Table 2). A significant difference between discrimination performance on trials reported as mislocalized with respect to trials reported as non-mislocalized was found for the perisaccadic interval (0, 20] and (40, 70], see Figures 5C,D,G and Table 2.

**Table 2:** **95% confidence intervals derived from the bootstrapped analysis on *p*(correct) and retinal distance data from 10 ms ISI experiment**.

ISI = 10 ms	p(correct) (%)				retinal distance (degrees/vis. angle)			
Mislocalized	[0.20, 0.45]	**[0.76, 0.87]**	[0.94, 0.99]	**[0.92, 0.98]**	[0.01, 0.02]	[0.04, 0.06]	**[0.97, 1.24]**	**[3.12, 3.47]**
Non-mislocalized	[0.28, 0.35]	**[0.36, 0.49]**	[0.95, 0.98]	**[0.29, 0.37]**	[0.01, 0.02]	[0.01, 0.02]	**[0.34, 0.76]**	**[1.24, 1.46]**
Perisaccadic interval	(−50, 0]	(0, 20]	(20, 40]	(40, 70]	(−50, 0]	(0, 20]	(20, 40]	(40, 70]

*Data reported for each time bin along saccade onset time. Significant differences between mislocalized and non-mislocalized trials in the *p*(correct) and retinal distance variables are reported in bold*.

In the case of retinal distance, participants showed a monotonic increase along the perisaccadic intervals from (−50, 0] ms till (40, 70] ms on trials in which the forward mask was reported as non-mislocalized as well as on trials in which the forward mask was reported as mislocalized (Table 2). A significant difference on trials reported as mislocalized with respect of trials reported as non-mislocalized was found for the perisaccadic interval (20, 40] and for the perisaccadic interval (40, 70] (Table 2), the pattern of retinal distance results differed with respect to those found for discrimination performance (see Figures 5C,D).

Retinal distance differed significantly between mislocalized and non-mislocalized masks during two perisaccadic intervals ((20, 40] ms and (40, 70] ms), hence the discrimination performance increase in mislocalized with respect to non-mislocalized masks found for the (40, 70] ms interval could be explained by a different retinal distance (Table 2). However this cannot be applied to the interval (0, 20] ms, where a performance increase for trials in which the mask was reported as mislocalized was observed, but retinal distance did not differ between mislocalized and non-mislocalized conditions (Table 2). Moreover, in the interval (20, 40] ms retinal distance differed significantly between mislocalized and non-mislocalized condition, but discrimination performance was not significantly different. Thus, retinal distance, mislocalization and discrimination performance were dissociated (see Figures 5C,D). Average proportion of trials where the mask was reported as mislocalized with respect to the target was: 16%, 40%, 75% and 20% for the (−50, 0], (0, 20], (20, 40] and (40, 70] perisaccadic interval, respectively.

### ISI = 0 ms

When asked to perform an eye movement while being presented with a forward masking sequence with an ISI = 0 ms, participants showed a discrimination performance increase along the perisaccadic intervals from (0, 20] and (20, 40] ms on trials were the forward mask was reported as mislocalized but not on trials where the forward mask was reported as non-mislocalized (Table 3).

**Table 3:** **95% confidence intervals derived from the bootstrapped analysis on *p*(correct) and retinal distance data from 0 ms ISI experiment**.

ISI = 0 ms	p(correct) (%)				retinal distance (degrees/vis. angle)			
Mislocalized	[0.17, 0.50]	**[0.63, 0.84]**	**[0.83, 0.98]**	[0.30, 0.75]	[0.01, 0.013]	[0.05, 0.08]	[0.85, 1.13]	**[1.16, 1.98]**
Non-mislocalized	[0.23, 0.36]	**[0.26, 0.42]**	**[0.15, 0.43]**	[0.26, 0.36]	[0.01, 0.012]	[0.02, 0.03]	[0.70, 1.09]	**[0.69, 0.87]**
Perisaccadic interval	(−50, 0]	(0, 20]	(20, 40]	(40, 70]	(−50, 0]	(0, 20]	(20, 40]	(40, 70]

*Data reported for each time bin along saccade onset time. Significant differences between mislocalized and non-mislocalized trials in the *p*(correct) and retinal distance variables are reported in bold*.

Again, the pattern of retinal distance results differed with respect to discrimination performance results. A significant difference between retinal distance on mislocalized vs. non-mislocalized condition was found for the perisaccadic interval (40, 70], see Figures 5E,F and Table 3.

Retinal distance differed significantly between mislocalized and non-mislocalized condition in one perisaccadic interval ((40, 70] ms), whereas discrimination performance increased in mislocalized with respect to non-mislocalized condition on the previous two perisaccadic intervals (0, 20] ms and (20, 40] ms, and not on the (40, 70] ms interval, see Figures 5E–G and Table 3. Average proportion of trials where the mask was reported as mislocalized with respect to the target was: 15%, 34%, 60% and 10% for the (−50, 0], (0, 20] (20, 40] and (40, 70] perisaccadic interval, respectively.

Experiment 3 results showed how retinal distance could at least partially explain discrimination performance when the ISI was set to 30 ms. However lowering the ISI to 10 ms lead to a partial dissociation between discrimination accuracy and retinal distance along the perisaccadic interval, while further lowering the ISI to 0 ms lead to a complete dissociation between discrimination accuracy and retinal distance along the perisaccadic interval.

## General Discussion

We measured the influence of saccadic eye movement on target discrimination performance on various conditions of a noise masking paradigm. In Experiment 1, we found that performing a saccade while presented with a train of rapid alternating masks and targets lead to improved discrimination with respect to stable fixation, in the absence of perceptual mislocalization. The largest discrimination advantage on saccade trials, of over 20% on average, was observed for a target-mask ISI of ~40 ms.

However, in Experiment 2 we established that masking effect on RSVP sequence were largely driven by the forward mask. We measured the time course of perisaccadic performance for a brief sandwich mask sequence in order to investigate the perisaccadic interval when target identity is resolved. Performance reached its peak while the eyes were moving towards the saccadic landing position, and this increase in performance could be largely explained by the retinal distance between the target and forward mask.

In Experiment 3 we investigated the role of perisaccadic unmasking on forward mask sequences, asking whether retinal distance also played a role or if perisaccadic mislocalization could improve performance on a forward masking sequence beyond the advantage expected due to increased retinal distance between forward mask and the target. In this experiment, participants were requested to report both the target identity and the relative position of mask and target after each trial. Three different ISIs were used: when ISI was set to 30 ms the perisaccadic performance pattern for mislocalized and non-mislocalized masks closely matched the retinal distance results. However for the shorter ISIs of 10 ms and 0 ms this was not the case. For the 10 ms ISI, we found a partial dissociation between retinal distance and perisaccadic performance on mislocalized and non-mislocalized masks along the perisaccadic interval. Moreover, there was a complete dissociation along the perisaccadic interval when the ISIs was set to 0 ms. Unlike backward masking sequences (De Pisapia et al., 2010), using a forward masking sequence, participants reported the mask (the first object presented on the screen) as mislocalized towards saccade landing position.

Given the overall pattern of results we can conclude that performing an eye movement over an RSVP sequence does improve discrimination performance. Our results indicate that the majority of the masking power in the RSVP sequence is driven by the forward mask. Moreover, the unmasking effect found using a forward masking sequence and an ISI compatible with the largest eye movement—fixation advantage found in the original RSVP sequence (~40 ms) can be accounted for by the retinal distance between forward mask and the target stimuli while the eyes are moving from starting to landing fixation point.

Interestingly, using a forward masking sequence and lowering the ISI to 10 ms and 0 ms, we found a partial (10 ms condition) and a complete (0 ms condition) dissociation between retinal distance and perisaccadic performance on mislocalized and non-mislocalized masks along the perisaccadic interval.

Neurophysiological studies showed that retinotopic neurons in the lateral intraparietal sulcus show a characteristic behavior around the onset of the eye movement: RF shifts spatially (Duhamel et al., 1992) from the current RF to the future RF (the position in space occupied by the RF after the completion of the eye movement). This shift anticipates the start of the actual eye movement and does not stop abruptly with the onset of the eye movement (Kusunoki and Goldberg, 2003). Instead, it evolves gradually during the perisaccadic interval, also when the eyes are actually moving towards saccade landing position.

We interpret our behavioral results on Experiment 3 with ISIs of 10 ms and 0 ms as a signature of the remapping signal gradually evolving while the eye are in motion from starting to landing fixation point. The dissociation between discrimination accuracy and mask—target retinal distance along the perisaccadic interval for these ISI levels speak in favor of this interpretation of the data. If the remapping signal would stop abruptly at the onset of the eye movement, then we would expect no discrimination accuracy advantage for mislocalized vs. non-mislocalized trials, along the perisaccadic interval, exceeding the trivial advantage given by larger forward mask—target stimuli retinal distance while the eyes are moving.

A simple response bias is an unlikely explanation for these results. It is conceivable that participants might be generally more prone to report the mask as being shifted towards target fixation point, when performing an eye movement. However, to accurately perform the task, participants had to be able to correctly report the shape of the target stimuli (3AFC), which cannot be accounted for by any response bias due to eye movement direction.

In Experiment 3 we used briefly presented targets and masks and asked participants to report whether they perceived the mask as mislocalized. It has been shown that participants perceive two briefly flashed stimuli around the perisaccadic interval in different spatial locations, depending on whether the stimuli are presented before the onset of the eye movement or while the eyes are moving towards saccadic landing position (Sogo and Osaka, 2002). We did not ask our participant to report the respective perceived location of the target and mask on each trial, but only whether the mask was perceived as mislocalized with respect to the target. Nonetheless we measured discrimination accuracy on a 3AFC, hence we could assess discrimination accuracy, irrespective of the perceived location, showing a clear advantage of trials reported as mislocalized compared to trials reported as non-mislocalized, an advantage that exceeds the benefit given by retinal distance alone.

Recent evidence shows that RFs of neurons in a FEF are compressed around the saccadic target during saccade preparation, right before the onset of an eye movement, whereas they remain centered in retinocentric space while fixating (Zirnsak et al., 2014). These results are consistent with previous behavioral reports, showing strong compression towards the saccadic target (Ross et al., 1997), and suggest that during saccade preparation the saccade target becomes over-represented compared to other portions of visual space. These results (Zirnsak et al., 2014) support the existence of a direct neural basis for the phenomenon of perceptual mislocalization and perisaccadic unmasking.

This view is also compatible with other behavioral findings, showing that covert attentional shifts precede every eye movement (Kowler et al., 1995; Deubel and Schneider, 1996). In the Deubel and Schneider study, for example, the authors found that targets presented at the saccade goal were more easily identified compared with targets presented 1.09° away from the saccade target, showing that shifts of attention are tightly coupled to the planned landing position.

## Conclusion

Our findings on Experiment 3 indicate that perisaccadic unmasking (due to perisaccadic mislocalization along the direction of the eye movement), can improve performance over a forward masking sequence. This performance improvement continues also after the eyes started moving towards the saccadic target, along the perisaccadic interval. Moreover, the observed performance improvement exceeded the benefit given solely by retinal distance.

These results are compatible with a view suggesting that an anticipatory shift drives RFs towards the saccadic target (Zirnsak et al., 2014). Data is indicative that this shift does not stop abruptly with the onset of the eye movement, a view compatible with data reported by Kusunoki and Goldberg (2003). Instead, the shift evolves continuously during the perisaccadic interval and is maintained also when the eyes are still moving towards the saccade landing position (see Figures 5C–F). Perisaccadic unmasking can alter the way in which a stimulus is perceived, both in terms of its spatial location and its visibility.

## Author Contributions

AF and DM made substantial, direct and intellectual contribution to the work, and approved it for publication.

## Funding

This research was supported by a European Research Council (ERC) grant (agreement no. 313658) awarded to David Melcher.

## Conflict of Interest Statement

The authors declare that the research was conducted in the absence of any commercial or financial relationships that could be construed as a potential conflict of interest.
